# Tuberculosis: opportunities and challenges for the 90–90–90 targets in HIV-infected children

**DOI:** 10.7448/IAS.18.7.20236

**Published:** 2015-12-02

**Authors:** Helena Rabie, Lisa Frigati, Anneke C Hesseling, Anthony J Garcia-Prats

**Affiliations:** 1 Division of Infectious Diseases, Department of Paediatrics and Child Health, Faculty of Medicine and Health Sciences, Stellenbosch University and Tygerberg Hospital, Tygerberg, South Africa; 2 Desmond Tutu TB Centre, Department of Paediatrics and Child Health, Faculty of Medicine and Health Sciences, Stellenbosch University, Tygerberg, South Africa

**Keywords:** tuberculosis, HIV, AIDS, children, infant, treatment, epidemiology, 90-90-90 target

## Abstract

**Introduction:**

In 2014 the Joint United Nations Programme on HIV/AIDS defined the ambitious 90–90–90 targets for 2020, in which 90% of people living with HIV must be diagnosed, 90% of those diagnosed should be on sustained therapy and 90% of those on therapy should have an undetectable viral load. Children are considered to be a key focus population for these targets. This review will highlight key components of the epidemiology, prevention and treatment of tuberculosis (TB) in HIV-infected children in the era of increasing access to antiretroviral therapy (ART) and their relation to the 90–90–90 targets.

**Discussion:**

The majority of HIV-infected children live in countries with a high burden of TB. In settings with a high burden of both diseases such as in sub-Saharan Africa, up to 57% of children diagnosed with and treated for TB are HIV-infected. TB results in substantial morbidity and mortality in HIV-infected children, so preventing TB and optimizing its treatment in HIV-infected children will be important to ensuring good long-term outcomes. Prevention of TB can be achieved by increasing access to ART to both children and adults, and appropriate provision of isoniazid preventative therapy. Co-treatment of HIV and TB is complicated by drug-drug interactions particularly due to the use of rifampicin; these may compromise virologic outcomes if appropriate corrective actions are not taken. There remain substantial operational challenges, and improved integration of paediatric TB and HIV services, including with antenatal and routine under-five care, is an important priority.

**Conclusions:**

TB may be an important barrier to achievement of the 90–90–90 targets, but specific attention to TB care in HIV-infected children may provide important opportunities to enhance the care of both TB and HIV in children.

## Introduction

An estimated 240,000 children were newly infected with HIV in 2013 despite scale-up of prevention of mother-to-child transmission (PMTCT), which resulted in a 40% reduction in perinatal transmission of HIV in 2011 compared to 2009 [1]. The majority of HIV-infected children (630,300) live in Africa, followed by Asia (54,100) and Latin America (26,400) [1]. Although 13.6 million HIV-infected people accessed combination antiretroviral therapy (ART) in 2014, this represents only 38% of adults and 24% of children with HIV. The regions with the lowest access to therapy are also in Africa (20 to 24%) and Asia (22 to 32%) [1]. In order to address these ongoing critical gaps in HIV care, the Joint United Nations Programme on HIV/AIDS defined the ambitious 90–90–90 targets to be achieved by 2020: 90% of persons living with HIV must be diagnosed, 90% of those diagnosed should be on sustained therapy and 90% of those on therapy should have an undetectable viral load. These targets were designed to stimulate rapid scale-up of sustainable and high quality HIV care, in order to prevent new HIV infections and to reduce HIV-associated morbidity and mortality. Adolescents and children were identified as key focus populations [2].

Tuberculosis (TB) remains a major cause of disease globally, with 9 million incident cases; 13% of these cases were HIV-infected. There were 1.5 million TB deaths in 2013, of which 360,000 were in HIV-infected persons. Although Asian countries (particularly China and India) account for the highest numbers of TB cases, all the countries with a TB incidence of more than 500 cases per 100,000 population are in Africa [3]. Models estimate that in 2010 there were 650,000 cases of TB disease in children and many more with latent infection [4]. In settings with a high burden of both diseases up to 56% of children treated for TB had HIV [5].

Given the close overlap of the TB and HIV epidemics, successful achievement of the 90–90–90 targets must specifically consider TB and its impact on HIV diagnosis, retention in care and attaining virologic suppression.

In this paper we discuss key aspects of the care of TB in HIV-infected children, highlighting threats and opportunities on the road to 90–90–90. We will discuss the epidemiology of TB-HIV co-infection, calling attention to the ongoing close interactions of these two diseases, as well as recent advances in the prevention of TB in HIV-infected children. We will describe developments in the treatment of TB and HIV and the implications of these for the reduction of morbidity and mortality in TB-HIV co-infected children. We will also focus on the emerging threat of drug-resistant TB.

## The epidemiology of TB-HIV co-infection

As described above, the TB and HIV epidemics have substantial overlap in their epidemiology. It is clear that Africa (particularly sub-Saharan Africa), South East Asia (Thailand and India) and Latin America carry the biggest burden of TB and HIV [1,6]. This has impacted children directly and through its impact on women of childbearing age. The HIV epidemic disproportionately increased the burden of TB in women of childbearing age [7]. One-third of TB deaths in women are in HIV-infected women [6]. Data from India confirms that where TB occurs in HIV-infected pregnant women, the infant is not only at risk for TB, but has a threefold higher risk of HIV acquisition and a fourfold increase in mortality [8–10].

### The risk of TB in HIV-infected children

HIV-infected and HIV-exposed infants are highly TB exposed in their households and local communities. In a South African trial of isoniazid preventive therapy (IPT), 10% of HIV-exposed infants had contact with a potential case of TB by 14 weeks of age [11].

In addition to frequent TB exposure, other conditions, including advanced immune suppression, poor nutritional status (stunting, wasting and reduced mid-upper arm circumference) and anaemia are associated with increased risk of TB in HIV-infected children [12]. The proportion of children with TB that are HIV co-infected varies from 5.8 to 56% depending on the setting [5].

Among South African infants with limited access to ART, up to 1595 cases per 100,000 population have been reported; this is more than 24 times the rate reported at the same time for HIV-uninfected South African infants [13]. The risk increases after six months of age in the absence of ART, with incidence rates of 6.2 to 18.1 cases per 100 person-years (age <6 months) and 9.4 to 46.3 cases per 100 person-years (age ≥6 months) reported in East Africa [14]. In other HIV-infected African children with poor access to ART, high risk of TB is also reported (more than 17 TB cases per 100 person-years) [15–18]. A review study of African post-mortem studies reports pulmonary TB in 8.3% of HIV-infected children [19].

In low-burden settings, the risk of TB in HIV-infected children is much less; however TB still occurs. Among children attending HIV services in the United Kingdom (between 1991 and 2006) and New York City (between 1989 and 1995) 3 to 5.5% had a diagnosis of TB at some point [20,21].

## TB prevention in HIV-infected children

TB co-infection negatively affects outcomes for HIV-infected patients, and there are challenges with TB diagnosis and co-treatment in HIV-infected children. These factors may all impact negatively on achieving 90% retention on ART and 90% virologic suppression; hence strategies to prevent TB in HIV-infected children are a priority for achieving the 90–90–90 targets.

### Antiretroviral therapy for HIV-infected children and adults

Increasing access to ART in paediatric cohorts decreases confirmed and probable TB. In the Children with HIV Early Antiretroviral Therapy (CHER) study, South African infants with deferred ART had 20 cases of TB per 100 person-years, compared to 8.3 cases per 100 person years in children receiving early ART [17]. Up to 33% of infants starting ART at a median age of eight months are already on TB therapy, indicating early infancy as a particular risk period and highlighting the importance of early initiation of ART [22]. A similar reduction in risk of TB in older children receiving ART has also been demonstrated in diverse settings in Africa, Asia and Latin America [15,16,18,22–28].

The first three months after initiation of ART represent a very high-risk period for hospitalization and death [29]. In addition, there is a substantial initial increase in the risk of TB, likely representing undetected TB, TB exposure at ART initiation or unmasking TB immune reconstitution inflammatory syndrome (IRIS) [27,30–33].

With increasing access to ART in adults, the risk of TB declines in adults as well as in HIV-infected and -uninfected children. In Johannesburg, South Africa, ART access in adults increased from 21.5% in 2005 to 68.2% in 2009 of those requiring ART. During the same period, TB in HIV-infected children declined from 1566.3 to 460.7 per 100,000 and in HIV-uninfected children from 18.7 to 11.0 per 100,000 [34]. If ART expands to cover 80% of adults living with HIV, TB incidence among all adults is projected to decline by 28 to 37% [35]. A reduction of adult TB cases, particularly among HIV-infected adults, would likely have an important impact on the risk of TB exposure, infection and disease among HIV-infected children.

Despite these clear benefits of ART, the risk of TB remains high in HIV-infected infants and children even when on ART. In South African infants with a 98% uptake of ART, those that were HIV-infected had a burden of 121 cases per 1000 years compared to 41 cases per 1000 child-years in HIV-exposed uninfected infants [36]. This highlights the importance of a multi-pronged strategy to address TB in HIV-infected children.

### BCG vaccination

Although Bacille Calmette Guérin (BCG), a live attenuated *Mycobacterium bovis* strain, is protective against disseminated TB, including meningitis in young children, it is less effective at preventing pulmonary TB [37]. BCG itself poses a risk to HIV-infected children, particularly those with severe immunosuppression and delayed initiation of ART. Disseminated BCG, a serious and potentially life-threatening complication of BCG vaccination, occurs in an estimated 992 (95% CI: 567 to 1495) per 100,000 HIV-infected infants [38]. In addition, infants initiating ART may develop IRIS related to BCG [39]. Given these concerns, the WHO recommended that BCG not be administered to persons who are confirmed HIV-infection [40]. As BCG is given at birth to healthy infants, this recommendation is problematic in settings where birth HIV DNA PCR is not routine, particularly as these are often the same settings with a high TB burden. Early infant diagnosis of HIV and early ART mitigates the risk of disseminated BCG and IRIS [41]. In a prospective study of 451 HIV-infected infants receiving early ART, none developed disseminated BCG disease; although BCG IRIS remained a problem, the risk was reduced threefold with early access to ART [39]. Rather than altering BCG vaccine delivery or managing IRIS, most countries are focusing on prevention of HIV transmission to children and access to ART [42].

### Isoniazid preventative therapy

Young age is an important risk factor for the development of TB disease after infection [43]. Treating latent *Mycobacterium tuberculosis* infection with isoniazid (INH) prevents disease [44,45]. Therefore the WHO recommends the provision of IPT for a minimum of six months to all children <5 years of age, and HIV-infected children of any age, with a documented infectious TB source case; this is an integral component of TB prevention in HIV-infected children [6].

Studies of pre-exposure and longer-term IPT in HIV-infected children have yielded conflicting evidence. In the only true pre-exposure prevention trial (IMPAACT P1041), 548 HIV-infected and 804 HIV-exposed uninfected infants between three and four months of age without TB exposure were randomized to INH versus placebo for 96 weeks with follow-up for another 96 weeks. The pre-exposure prevention did not reduce the TB incidence in either HIV-exposed-uninfected or HIV-infected infants [36]. An earlier placebo controlled study of IPT for all HIV-infected children enrolled older children with delayed access to ART, including those with prior TB or TB exposure. This study showed a significant reduction in TB and all-cause mortality (9.9 and 16% in the placebo arm vs. 3.8 and 8% in the IPT arm). Subsequent analysis of this cohort illustrated that combining IPT with ART was more effective than either strategy alone to prevent TB [16,46]. In 2014, the WHO recommended routine pre-exposure IPT for six months in all HIV-infected children older than 12 months without evidence of TB disease [6]. Children with suggestive symptoms (poor weight gain, fever and current cough) or with a history of TB exposure should be investigated for TB prior to IPT initiation. Diagnostic challenges in HIV-infected children remain a reality, especially if there is no TB contact but suggestive symptoms. The WHO guidelines also recommend that children treated for TB with a good response should receive six months of INH post–TB treatment completion; however, there is little evidence for this recommendation.

There are substantial challenges in IPT implementation, including health system weakness and lack of availability of child-friendly INH formulations. Missed opportunities for chemoprophylaxis are well documented. A community-based study from South Africa found that only 21% of eligible children received IPT and that the lack of specific tools to ensure implementation possibly contributed to low uptake [47]. Poor adherence to IPT remains a challenge [48]. Health care worker training and introduction of specific documentation such as cards and registers, as well as contact tracing, increased uptake of IPT from 16 to 61% [49]. Integration of TB and HIV services may also increase IPT completion [50].

In HIV-uninfected children, adherence is improved by using rifamycins, which require shorter courses [51]. This is not an option for HIV-infected children on ART or HIV-exposed infants on extended nevirapine (NVP) prophylaxis for HIV prevention, as rifamycins have important drug-drug interactions with a number of antiretrovirals. Twelve doses of rifapentine-INH, recently shown to be effective in HIV-uninfected children, was not studied in HIV-infected children [52].

There is no published data in HIV-infected children on the benefit of tuberculin skin testing or other tests of infection, such as the interferon gamma release assays, to help target pre-exposure IPT. Data in HIV-infected adults suggests that TST-positive adults benefit the most from IPT [53]. No TB prevention studies have specifically targeted adolescents. However, the risk of TB increases during adolescence [43], and HIV-infected adolescents with TB exposure or infection would potentially benefit considerably from IPT. Drug resistance in TB source cases may be a major factor in children failing IPT. In the IMPAACT P1041 trial, children developing culture-confirmed TB on INH did not have INH mono-resistance, but rather multidrug-resistant (MDR) TB [54]. Obtaining a thorough history of the potential contacts prior to IPT initiation is important; if a child on IPT develops TB, the contacts should be reviewed again for resistance and clinically relevant specimens should be taken for culture and susceptibility testing prior to starting TB treatment.

### Other TB prevention opportunities

There are a number of other additional opportunities for preventing TB in HIV-infected children. Temporal associations between TB, influenza and pneumococcal disease in children suggest a complex interaction between these infections. An increase in TB diagnoses following approximately three months after an influenza epidemic in a high burden setting suggests a potential role for the influenza vaccine in reducing the TB burden [55]. A study of TB after introduction of conjugated pneumococcal vaccine showed that culture-confirmed TB was 47% less in vaccinated compared to unvaccinated HIV-infected children [56]. Cotrimoxazole appears to have anti-mycobacterial activity [57] and conflicting evidence suggests cotrimoxazole prophylaxis may reduce incident TB in HIV-infected adults [58,59]. In a trial of 758 HIV-infected children on ART randomized to prolonged cotrimoxazole despite immune reconstitution, there were fewer incident cases of TB, although numbers were small [60]. These strategies, in addition to reducing their targeted disease, may have the benefit of reducing TB, adding to the urgency of their implementation in HIV-infected children. In the absence of a highly effective TB vaccine, a multi-pronged approach to TB prevention is likely to be the most successful and is important in enabling achievement of the 90–90–90 targets.

## Outcomes among TB-HIV co-infected children

Prior to wide access to ART, high mortality of co-infected children was reported. A study from South Africa documented a 10 times higher mortality in TB-HIV co-infected children than in HIV-uninfected children with TB [12]. Children with TB-HIV co-infection who died often had additional pulmonary infections on post-mortem [61]. With access to ART, better outcomes are reported, particularly for children who develop TB while on ART for more than six months; however, mortality does occur especially in the first two months of ART. A retrospective observational study from South Africa reported 10% mortality in TB-HIV co-infected children less than two years of age [18,22]. Deaths may be due to TB, other HIV complications or IRIS. The effect of TB co-infection on mortality thus affects the achievement of 90% retention on ART.

### Virologic outcomes in children co-treated with rifampicin

Concomitant TB therapy may be a significant risk factor for virologic failure in children who are co-treated, impacting the target of 90% of children on ART being virologically suppressed. Viral load is not available in all settings where there is a high TB burden. In settings where the protease inhibitor (PI) lopinavir/ritonavir (4:1) LPV/r is the preferred initial therapy in children<3 years and super-boosting is used, high levels of viral suppression are reported for children, regardless of co-treatment. However the rates of suppression at 6 and 12 months are lower than those of children not requiring co-treatment [62–64]. Studies of children failing LPV-based regimens have shown accumulation of PI mutations in 10% of children tested a median of 21 months after initiation of therapy [65]. Although concurrent TB therapy may be a risk factor for virologic failure, it was not necessarily a risk factor for LPV resistance [67]. A likely explanation is the robust resistance profile of LPV; however great care should be taken to ensure dosing accuracy and appropriate addition of ritonavir (RTV). RTV at full dose should not be used as therapy, as it is associated with the highest risk of failure and PI resistance [62,66].

No difference in virologic suppression has been seen with efavirenz (EFV)-based therapies at month 12 [64]. There is limited data on the routine virologic outcomes of children treated with NVP. However, poorer virologic outcomes are reported in several cohort studies when compared to EFV, regardless of TB therapy [67,68].

## Treatment

Optimizing the co-treatment of TB and HIV is important to ensuring the targeted 90% treatment success in HIV-infected children.

In low resource settings, TB treatment initiation is as frequent a cause of ART regimen changes as adverse effects [69]. When choosing an ART regimen, apart from drug interactions, clinicians should also consider prior ART, available drugs and formulations, age and weight. For children on ART, clinicians must also consider duration on therapy, adherence to therapy and the probability of therapeutic failure.

### First-line TB medications and drug-susceptible TB

In 2010, the WHO recommended higher doses of the first-line anti-TB drugs in all children [70]. This recommendation was based on an extensive review of evidence demonstrating low exposure with the previously recommended doses. These changes introduced practical challenges, as the ratio of RMP, INH and pyrazinamide (PZA) in the existing paediatric fixed-dose combination (FDC) formulations does not allow for easy dosing within the new recommendations [71].

Among 20 children younger than two years receiving the newly recommended doses of INH, RMP and PZA, nearly all children had maximum serum concentrations of these medications above target levels. HIV-infected children in this study (*n=*5) had a significantly lower C_max_ and T_max_ on PZA 35 mg/kg, but no difference in total exposure; the small sample size may have limited the ability to detect other differences [72]. In 31 South African children younger than 10 years, the majority receiving the new WHO-recommended doses of the first-line TB medications, two-hour target concentrations were attained for RMP in only 2/31 (6%), for INH in 20/31 (65%), for PZA in 17/31 (55%) and for ethambutol (EMB) in 2/13 (15%); HIV infection (*n=*7 children) was associated with a low two-hour INH concentration [73]. It is not clear whether the reduced TB drug concentrations reported in these studies are related to interactions with ART or the direct effects of HIV infection; neither study clearly describes the ART regimens in the included HIV-infected children. Studies of the first-line TB medications in children are ongoing (NCT01637558 and NCT01687504).

Despite limited evidence, the WHO recommends the addition of EMB to the intensive phase of TB treatment in children with HIV, extensive disease or in settings with a high prevalence of INH resistance [6]. The rationale for this is that EMB may protect against the acquisition of RMP resistance in children with existing INH resistance; acquired RMP resistance in children has been described, although the actual risk is not known [74]. Additional data on the benefit, risk and programmatic implications of this recommendation for HIV-infected children is needed. An extensive review of the literature identified very few reports of EMB-associated ophthalmologic toxicity in children [75]. Recently in a study of a small number of children treated with EMB, newer technology identified reversible ophthalmological complications attributed to EMB. EMB should continue to be used when indicated, but this risk deserves further evaluation [76].

### Second-line TB medications

Despite common use there is little data on the pharmacokinetics of second-line TB medications in children [77]. The currently recommended doses of ofloxacin, levofloxacin and moxifloxacin result in drug exposures considerably lower than in adults, and moxifloxacin exposure is significantly lower in HIV-infected compared to HIV-uninfected children [78,79]. It is not clear if this is due to poor absorption or a potential drug interaction with RTV [79,80]. In HIV-infected adults, co-treatment with EFV results in a more than 30% reduction in para-aminosalicylic acid exposure [81]. There are no recommendations to alter doses of any of the second-line TB medications or ART in co-treated children, but additional data is urgently needed.

HIV infection may be a risk factor for adverse events among children treated for MDR-TB. A higher risk of ethionamide-induced hypothyroidism has been shown compared to HIV-uninfected children [82]. Linezolid, used in children with extensively drug-resistant TB, may have additive adverse effects with nucleoside reverse transcriptase inhibitors (NRTIs) due to mitochondrial protein synthesis inhibition and should be used with caution in HIV-infected children [83].

### Novel TB medications

Two novel TB medications, the ATP-synthase inhibitor bedaquiline and the nitroimidazole delamanid are increasingly used in adults [84,85]. Phase 1 and 2 studies of delamanid in children (NCT01856634, NCT01859923) have begun enrolment, and studies of bedaquiline in children are planned. Current studies exclude HIV-infected children but future studies will include them. Metabolism of delamanid is partially mediated by the cytochrome p450 (CYP) 3A4 enzyme, but healthy volunteer studies indicated no clinically significant drug-drug interactions with ART [85]. Bedaquiline is metabolized by CYP3A4 isoenzyme; co-administration with the CYP3A4 inducer EFV reduces exposures of bedaquiline and its main metabolite, M2, by roughly 50%. Co-administration with lopinavir, an inhibitor of CYP3A4, reduces clearance by 35% for bedaquiline and by 58% for M2, resulting in two- to threefold higher exposures of bedaquiline and M2. There is no clinically significant interaction between bedaquiline and NVP [86–88]. Data on the pharmacokinetics and safety of bedaquiline and delamanid in HIV-infected children will be important to ensure these medications are accessible and able to be safely used in these children.

### Deciding on the appropriate ART in children with TB

Drug-drug interactions between TB medication, especially rifamycins, and ART are a major concern, as they may influence the virologic outcomes of co-treated patients, leading to HIV treatment failure and increasing the risk of ART resistance, potentially limiting future therapy. The drug interactions between NRTIs and RMP are not considered clinically significant; however there are significant interactions with non-nucleoside reverse transcriptase inhibitors (NNRTIs), PI and integrase inhibitors. For some of these interactions, there is no paediatric data.

### Protease inhibitors

LPV/r is superior to NVP in young children regardless of previous NVP or EFV exposure through PMTCT [89,90]. LPV/r is now recommended as first line in most children <3 years of age [91]. Given its more prominent role, drug-drug interactions with TB medications are important. LPV/r concentrations are reduced by RMP CYP3A4 induction and through changes in the p-glycoprotein expression [92]. Adding RTV to alter the ratio from 1:4 to 1:1 (so called super-boosting) was shown to overcome this RMP effect in a study of 15 children (median age 16 months, median weight 8.6 kg). The median C_max_ and AUC_0–12_ were lower than in controls, but the C_min_ (the target for efficacy) was greater than the minimum recommended. Children tolerated the strategy and two cases had mild alanine transaminase elevation that did not require therapy interruption [93]. Preliminary data from an ongoing study of a large cohort of young TB-HIV co-treated South Africans receiving super-boosting (NCT02348177) confirms this finding [94].

In adults, giving double the dose of LPV/r was found to have acceptable pharmacokinetic and less toxicity [95]. However, of 20 children (median weight 9.1 kg, median age 1.2 years) receiving double-dose LPV/r (460/115 mg/m^2^ twice), 80% did not achieve a target morning trough of 1 mg/L [96]. Explanations include characteristics of the formulation as well as drug absorption and metabolism. A modelling study suggests that overcoming the interaction with RMP using the LPV/r 4:1 solution with twice daily administration will require such high doses that there may be adverse events, whereas an eight-hourly dosing regimen may overcome the drug interaction; a study of this is ongoing [97]. The individual RTV formulation requires refrigeration for storage and has a short shelf life, complicating its use in resource-limited settings, and when only used for super-boosting may be a challenge for supply chains to maintain continuous widespread availability. Additionally, it is poorly palatable and may be problematic to administer to children. In settings increasingly utilizing task-shifting and relying on nurses for ART provision, these complicated drug-drug interactions between LPV/r and TB medication may be a barrier to a super-boosting strategy. The optimal approach to ART in TB-HIV co-infected children may need to be considered by each high-burden country depending on these contextual issues, and improved options are needed. In order to facilitate access to LPV/r, mini-tablets were developed and are now licensed by FDA.

Rifabutin, which does not affect PI concentrations, is also problematic. Rifabutin is metabolized by CYP3A4 and dose adjustments of rifabutin are needed if co-treating with PIs; however pharmacokinetic data in children is limited. In six children treated with rifabutin 5 mg/kg three times per week and with LPV/r, severe transient neutropenia and insufficient rifabutin exposure was observed [98]. Using rifabutin in a public health programme is currently not possible due to lack of data in co-treated children, complex two-way interactions, cost and lack of an FDC.

There is a lack of paediatric data for boosted atazanavir (ATV) and darunavir (DRV), which also both interact with RMP. In adult volunteers, double doses of ATV with RMP did not correct the ATV exposure but did not cause substantial toxicity [99]. For DRV, modelling of adult data suggests that dose increases of DRV and RTV (800/100 mg and 1200/150 mg twice a day) both may overcome the RMP induction; the usual adult dose is 800/100 mg daily for naïve patients older than 12 years of age [100]. This dose increase may, however, cause adverse effects and there are no published data studying this approach. The interaction and optimal PI treatment strategy in children with TB is a critical area for future research given the importance of these medications in ART regimens.

### Non-nucleoside reverse transcriptase inhibitors

EFV is used in older children and is superior to NVP [67]. Recent data suggests that no adjustment of EFV dose is required when RMP is used [101,102]. Previously a lack of data in dosing prevented EFV use in children younger than three years. Though it is now licensed for this age, experts recommend CYP2B6 genotype prior to EFV initiation in this age group [103]. Using EFV in this age group with prior NNRTI exposure has not been studied and data on co-treatment with RMP-containing TB regimens are not available.

NVP is a commonly used NNRTI in low resource settings, where it is incorporated in easy-to-use and well-tolerated FDCs. There are reports of adequate exposure in RMP co-treatment but larger co-treatment studies found significant under-dosing even if NVP was given at more than the standard recommended dose [104–106]. The period of initiating NVP-containing ART using daily NVP for 14 days may be particularly risky in children who are also on RMP, as NVP concentrations may be low; avoiding the induction dosing in all children younger than two years of age has been suggested [107]. Where NVP use cannot be avoided in TB co-treated children, a dose of 200 mg/m^2^/dose twice daily should be used [91]. Switching children with TB from NVP to EFV should be considered wherever possible. Lastly, children taking NVP as PMTCT, where mothers are on RMP or where the infants require RMP-TB therapy, may have low NVP concentrations [108]. These low concentrations may reduce the efficacy of NVP in preventing HIV.

Switching suppressed children from LPV/r to EFV is a strategy studied in children without TB [109]. Although prospective studies have not been performed, a switch to EFV can be considered if children develop TB. If at all possible, viral load monitoring should be done to detect early failure, in which case further action must be taken.

Etravirine is not typically available in low resource settings and is not approved for use in children less than six years of age. There are no adult studies assessing the interaction of RMP and etravirine, with the exception of case reports, which confirm a significant reduction in plasma levels, which did not impact virologic suppression [110].

Based on results of the Antiretroviral Research for Watoto (ARROW) study, the WHO recommends triple NRTIs as a preferred strategy in children age <3 years requiring RMP co-treatment. In ARROW, three NRTIs were studied as an intensive induction and a maintenance strategy when combined with NNRTIs. After 48 weeks of initial therapy that contained a NNRTI children were randomized to a three-drug NRTI maintenance regimen or to remain on conventional therapy. Fifty-nine of the 1143 children required a drug alteration for TB that included stopping or replacing NVP. Children on EFV did not require drug switch. Triple NRTI was effective at 36 weeks, but not at 144 weeks. The advantage of this strategy is that, although viral suppression is inferior, the risk of progressive NNRTI and PI resistance was avoided and complicated drug-drug interactions and logistic complications were easier to manage [69]. Where PIs are available, limited NRTI resistance may not compromise future suppression. However, this strategy could be problematic in very ill children where viral control and immune recovery is essential to improve the health of the child.

### Integrase inhibitors and other issues

Integrase inhibitors are an increasingly important class of ART, particularly as a key component of third-line regimens. Raltegravir and dolutegravir are not metabolized by the CYP P450 enzymes but are metabolized through the liver by uridine 5-diphospho (UDP)-glucuro-nosyltransferase 1A1, an enzyme that is also induced by RMP. Healthy volunteer studies showed a significant reduction in raltegravir exposure with RMP co-treatment [111]. In a prospective study comparing standard and double-dose raltegravir with EFV in ART-naïve adults requiring RMP, no significant difference was found in virologic suppression at 24 weeks between the standard and double-dose raltegravir groups, with both groups similar to the EFV group [111]. There is no published data in children; however an ongoing study is assessing the effective dosing, safety and tolerance of children co-treated with raltegravir and RMP (NCT01751568).

Adult healthy volunteer studies assessing the effect of RMP on dolutegravir suggest that doubling the dolutegravir dose is needed if there is co-treatment with RMP [112].

Children needing third-line therapy and co-treatment with RMP-containing TB therapy may benefit from a holding triple NRTI strategy. This may be a good option if the children have a preserved CD4 count and are clinically stable. Where children require a suppressive regimen urgently, consideration should be given to changing to a non-rifamycin-containing TB regimen. Rifabutin also has less substantial interactions, but dose adjustment of rifabutin and ART may still be needed. Rifapentine is not recommended, as there may be a risk of RMP-monoresistant TB developing [113]. Fluoroquinolone-based TB regimens could be considered, but have not been prospectively studied in this context

### Approach to timing of ART initiation

The timing of ART initiation in adults with TB has been extensively studied. Delaying therapy in adults with a CD4 count of <200 cells/mL has been associated with poor virologic and clinical outcomes [112]. The timing of ART initiation in children with TB has not been studied prospectively. In observational paediatric cohorts, delaying therapy for up to two months was not associated with an increased risk of mortality or poorer virologic response [114]. Whether longer delay in older children and adolescents with good CD4 counts and minimal TB disease is acceptable is not known.

TB meningitis (TBM) is an exception regardless of CD4 count, since clinical deterioration due to paradoxical IRIS can be devastating. In the adult literature, IRIS is associated with more disseminated forms of the disease and positive culture in the cerebrospinal fluid. It is common practice to delay ART four to six weeks in TBM [115]. Although there are fewer data in children, case series confirm the high morbidity associated with paradoxical IRIS [116]. There are no prospective data in children and it is important to keep in mind that the outcomes in patients with TBM are also determined by the severity of meningitis. Only 20% of HIV-uninfected children are neurologically normal after full TBM treatment [117].

If children are already on ART, appropriate anti-TB therapy should be started as soon as the appropriate diagnostic testing has been performed. ART should be adapted and the possibility of virologic and or immunological failure must be considered and appropriately investigated.

## Drug-resistant TB

MDR-TB (resistant to INH and RMP) is a growing health threat with an estimated 480,000 cases occurring in 2013; 9% of these cases also had additional resistance to a fluoroquinolone and/or a second-line injectable medication [3]. Despite limited data on the burden of MDR-TB in children, a 2010 model estimates a burden of 32,000 cases. Children typically have transmitted or primary MDR-TB. Among childhood MDR-TB cases in South Africa, HIV infection was reported in 53.6, 77 and 22% in Johannesburg, Kwa-Zulu Natal and Cape Town [118–120]. HIV infection was independently associated with prevalent TB disease among child household contacts of adult MDR-TB cases and is associated with poorer outcome in child MDR-TB household contacts on MDR preventive therapy [121] HIV-infected children were also older and had more severe disease [122]. A recent review of adults and children with HIV and MDR-TB showed 83.4% treatment success in children [123]. It is becoming increasingly important to ensure that diagnostic, prevention and treatment strategies for MDR-TB are developed for HIV-infected children.

## Operational issues

Initially TB and HIV services were introduced as vertical programmes, but now strategies to integrate these services and strengthen the linkage to antenatal care are essential to improving diagnosis and access to care and treatment. HIV testing remains the entry point for HIV care. Ensuring that children diagnosed with TB are tested for HIV is absolutely essential. Given the high risk of HIV co-infection in children with TB in many settings, routine HIV testing of all children receiving TB treatment is an important opportunity to increase HIV diagnosis and thus achievement of 90% of HIV-infected persons knowing their status.

The diagnosis of TB in all children remains challenging. A study of paediatric ART programmes in diverse resource-limited settings showed that, although sputum microscopy and CXR were available in all programmes, they were only used in 86 and 52% of TB diagnoses. Xpert MTB/RIF was only used in 8% of TB diagnoses and mycobacterial culture in 17%. Eighty-six percent of sites provided access to TB treatment, but 30% never provided IPT to children [124]. Adult contact tracing and access to IPT remains a vital component of care in HIV-infected children regardless of the ART therapy. This undertaking starts with taking the appropriate history at each health care contact. HIV-infected children who have defaulted care may be picked up and re-enter care at TB services.

Screening of pregnant women for both HIV and TB may improve access not only to PMTCT, but also to IPT. Integrating TB services into antenatal care of HIV-infected women may also be key to preventing TB in young infants [125]. In Table 1 we highlight the components of care that require linkage and integration. Health care providers in all these settings need competency in all these aspects. Health system strengthening aimed at meeting the 90–90–90 targets will need to consider TB services and may have beneficial impacts for TB care in children as well.

**Table 1:** Components of care that require linkage and integration to deliver tuberculosis preventative and treatment services to HIV-infected children

	Tuberculosis services	HIV services	Antenatal care	General healthcare/IMCI
Screen for TB contact and link to IPT		✓		✓
Trace contacts	✓	✓	✓	
Screen for TB		✓	✓	✓
Diagnose TB	✓	✓	✓	✓
Diagnose HIV	✓	✓	✓	✓
Treat TB/link to TB care	✓	✓	✓	✓
Provide ART link to ART care	✓		✓	✓
Adapt drug choices to accommodate rifamycin	✓	✓		✓
Ensure adherence to all therapies	✓	✓		✓

IMCI: Integrated Management of Childhood Illness; TB: tuberculosis; IPT: isoniazid preventative therapy; ART: antiretroviral therapy.

## Conclusions

The TB and HIV epidemics remain closely interlinked and TB is still an important opportunistic infection in HIV-infected children, with substantial mortality and morbidity and with co-treatment possibly affecting HIV outcomes. The growing burden of drug-resistant TB poses new challenges. Increasing ART access has the potential to greatly impact the TB epidemic in settings with high dual burden. The reduction in adult cases will reduce TB infection to both HIV-infected and -uninfected infants and children. Early ART in HIV-infected children will further reduce the burden of TB in these children. The provision of IPT should be strengthened, and innovative prevention strategies such as influenza and pneumococcal vaccination and continuing co-trimoxazole should be explored. Treating these diseases simultaneously presents challenges with regards to choosing the most appropriate regimens and ensuring that medications are available in all settings and easy for children to adhere to. There is a synergy between working towards the 90–90–90 targets and improving TB diagnostic and treatment programmes. Efforts to meet the 90–90–90 targets for both adults and children may well have a profound impact on the burden of childhood TB, while improved prevention, diagnosis and treatment of TB in co-infected children, as well as strengthening and integration of TB-HIV programmes, will be important if the 90–90–90 targets are to be achieved.
